# Assessment of LASER- induced precipitation of MTA-nanoparticles on root canal dentin surface

**DOI:** 10.1038/s41405-025-00322-y

**Published:** 2025-04-19

**Authors:** Mohammed Hamdi Atteia, Abeer Ahmed Saba, Eman M. Fouad

**Affiliations:** 1 Department of Endodontics, Collage of Oral and Dental Surgery- Misr University for Science and Technology(MUST), P.O.Box77, Giza, Egypt; 2 Department of Endodontics, Faculty of Dentistry- Cairo University, Cairo, Egypt; 3 Department of Endodontics-Collage of Oral and Dental Surgery- Misr University for Science and Technology(MUST), P.O.Box77, Giza, Egypt

**Keywords:** Root canal treatment, Mineral trioxide aggregate

## Abstract

**Aim of the study:**

This study aims to evaluate the effectiveness of a 980-nm diode laser in inducing mineral trioxide aggregate (MTA) nanoparticle precipitation on root canal dentin surfaces for dentinal coverage.

**Materials and methods:**

Sixty mature single-rooted teeth were decoronated at a fixed length of 16 mm and instrumented to size #40/0.04. Canals were filled with either distilled water or nanoMTA suspension and randomly divided based on the treatment modality into: G I and G II flooded with distilled water and laser irradiated at 2 Watt and 4 Watt respectively, GIII and GIV flooded with nanoMTA suspension and laser irradiated at 2 Watt and 4 Watt respectively, G V, flooded with nanoMTA suspension without laser irradiation, and G VI flooded with distilled water without laser irradiation. All samples were longitudinally split and scanned by environmental scanning electron microscopy (ESEM) to evaluate dentinal tubule (DT) occlusion and MTA surface precipitation. Image J analysis software was used to quantify open DTs, while a scoring system assessed dentine coverage.

**Results:**

Laser irradiation significantly enhanced nanoMTA precipitation and dentinal tubule occlusion. The highest dentinal surface coverage, indicated by the lowest pixel percentage, was in laser-irradiated nanoMTA suspension groups G IV (3.4 ± 3.1) and G III (16.7 ± 3), while the lowest coverage was in the non-irradiated saline group G VI (53.4 ± 9.6) (*p* < 0.05). Median dentinal coverage scores were also highest in G III and G IV (both = 4). G VI showed the least dentinal occlusion, with a statistically significant difference from other groups (*p* < 0.05).

**Conclusion:**

The 980-nm diode laser effectively enhances MTA nanoparticle precipitation on root canal surfaces, improving dentinal tubule occlusion and sealing potential. Further research is warranted to optimize laser parameters, MTA suspension ratios and to assess clinical outcomes.

## Background

Root canal treatment plays a pivotal role in preserving pulpal and/or periapical affected natural teeth, with success hinging on an effective seal after chemo-mechanical canal preparation. A silicate-based sealing material has become popular for this purpose due to its unique sealing properties. Mineral trioxide aggregate (MTA) which was first introduced by Torabinejad in early 1990s drives its chemical composition from Portland cement [1]. MTA primarily consists of tricalcium silicate (3CaO∙SiO_2_) and dicalcium silicate (2CaO∙SiO_2_) as its main hydraulic components, along with smaller amounts of tricalcium aluminate (3CaO∙Al_2_O_3_), calcium sulfate (CaSO_4_), and bismuth oxide (Bi_2_O_3_) as a radiopacifier [2]. Previous literature has reported that MTA is composed of approximately 80% Portland cement and 20% bismuth oxide [3]. It also comprises hydrophilic particles that induce a setting reaction under conditions of elevated humidity [4].

Due to the notable attributes of MTA as; high sealing properties, high biocompatibility and being bioactive [5], it has been increasingly favored for pulp capping, pulpotomy [4], apexification and as root end filling material [6]. Recently, it has been applied as a root canal sealer [7] and as an obturating material [8]. Despite recent evidence that advocate caution when using MTA as a root canal sealer [9], MTA can promote the regeneration of the periodontal ligament and the formation of cementum within the root canal space and accessory canals, thus effectively closing the leeway spaces that can result in the treatment failure [10].

Nanotechnology, with its nano-scale particle sizes, enhances surface area and reactivity, significantly impacting the material’s physical and chemical properties. These changes also affect biological responses and improve biocompatibility [11]. Nano MTA revealed good adaptability and sealing ability in the literature [12]. Moreover, Nano scaled MTA particles in nano white MTA demonstrated better repair, higher rates of bone formation and reduced inflammatory response compared to the conventional formulation [13].

Laser technology holds significant potential in material sciences. When laser energy is absorbed by target material, rapid and localized heating that triggers a series of photo-thermal reactions occur in a highly selective manner [14]. Subsequent sintering steps, involving melting and coalescence between nanoparticles, contribute to the creation of interconnected sintered films [15]. The current body of literature highlights the potential of selective laser sintering (SLS) as an effective technique for producing functional layers on heat-sensitive substrates [15].

Accumulating evidence supports the efficiency of heat to allow solid particles sintering, where particles bonding was facilitated at the atomic scale and could be evident by the SEM [16]. For instance, the laser has the potential to selectively and locally elevate temperatures, thereby inducing solid-phase sintering. A previous study by Pan et al., in which a 248 nm KrF excimer laser beam induced TiO_2_ nanoparticles sintering, where the spray-deposited nanoparticles were annealed in successive layers [17]. Similarly, dye solar cells were fabricated with nanocrystalline TiO_2_ films that were sintered using a pulsed ultraviolet laser, with the average laser output power ranging from 1 W to 7 W [18]. Likewise, the potential of selective laser sintering of silver nanoparticles in solution to fabricate microstructures [19].

Unfortunately, the evidence gathered was based on and intended for industrial and engineering purposes, with limited exploration in clinical settings, particularly regarding laser-induced dental biomaterial precipitation and sintering. Effective root canal sealing is essential for long-term success, as unsealed dentinal tubules can lead to bacterial ingress and reinfection. While MTA offers excellent biocompatibility and sealing properties, controlled precipitation onto dentinal surfaces could further enhance its effectiveness. Conventional methods rely on MTA’s intrinsic setting, which may not ensure optimal coverage. Laser-induced precipitation and sintering offer a novel approach to enhance MTA adhesion and dentinal interaction, potentially reinforcing the seal before final obturation.

Given the scarcity of research on using laser power to induce MTA nanoparticle precipitation and selective sintering within root canals, further investigation is warranted. This study aims to evaluate the potential of a 980-nm diode laser to enhance MTA nanoparticle precipitation onto root canal dentin surfaces, improving dentinal coverage. The null hypothesis states that the 980-nm diode laser does not significantly induce MTA nanoparticles precipitation compared to non-irradiated samples.

## Materials and methods

### Sample-size calculation & ethical approval

Sample size was calculated with the help of G-Power 3.1.9.7 program, based on a previous study [20]. The power analysis was conducted to achieve a statistical power of 0.80 with an alpha level set at 0.05. This determined the minimum number of 10 samples per group. The present study was reviewed and approved by the Institutional Review Board Organization IORG0010868, Faculty of Oral and Dental Medicine, Ahram Canadian University under the Number: IRB00012891#56.

### Canal preparation

Sixty recently extracted mature single-rooted teeth were decapitated at the cemento-enamel junction (CEJ) and the root portions were set at fixed length of 16 mm, Canals were instrumented to an apical size of #40/0.04 using K3 rotary NiTi files (SybronEndo 1332 S. Lone Hill Avenue, Glendora, CA 91740-5339 USA). Canals were instrumented in presence of EDTA gel lubricant (MD-ChelCream, META-BIOMED, Korea) and frequently irrigated with 2 ml of 2.5% NaOCl between each file. A final irrigation protocol involved canal irrigation with 1 ml of 17% EDTA for 60 s, followed by 5 ml of distilled water irrigation as a final flush to remove the residual effect of any chemicals.

### Nano-MTA preparation and characterization

The MTA nanoparticles were produced and the composition mentained by NanoTech (NanoTech for Photo-Electronics, Giza, Egypt). The primary steps in the processing route were as follows: First, calcium nitrate and then aluminum nitrate were dissolved in 180 ml of water while being magnetically stirred until a clear solution was formed. Subsequently, tetraethyl orthosilicate (TEOS) was added and hydrolyzed. The solution was continuously stirred to promote water evaporation and accelerate the polycondensation reaction, leading to the formation of a viscous gel. This gel was then dried at 120 °C for 420 h, resulting in a white powder as the final product [3, 21, 22]. The transmission scanning electron microscope revealed a spherical particle shape with average size less than 100 nm.

### Preparation of MTA suspension

A suspension was prepared by adding half gram of white nano MTA powder particles to 6 ml of distilled water, in the ratio of 1:12. The solution was freshly prepared for every five minutes of laser application in a test tube, manually agitated for 10 s and left in an upright position for 30 s. The middle layer of the suspension was then aspirated in a plastic disposable syringe with hypodermic needle of #25 gauge.

### Samples classification

Root samples were divided randomly through computerized random number generator (Microsoft Excel software) into 6 groups (*n* = 10), where canals were flooded with 1 ml of solution, namely, distilled water or nano-MTA suspension.

In groups G I and G II, the root samples were flooded with distilled water. Subsequently, each group samples were diode-laser-irradiated with a power setting of either 2-W (G I) or 4-W (G II), respectively. In groups GIII and GIV, the root samples were flooded with nano-MTA suspension. Subsequently, each group samples were diode-laser-irradiated with a power setting of either 2-W (G III) or 4-W (G IV), respectively. Samples of G V were flooded with nano-MTA-suspension, whereas samples of GVI were flooded with distilled water (negative control), both without subsequent laser irradiation. Samples classification is shown in Table 1.

**Table 1:** Classification of teeth samples according to treatment modality.

Group code	Treatment
G I	flooded with distilled water, irradiated with 2 W diode.
G II	flooded with distilled water, irradiated with 4 W diode.
G III	flooded with MTA suspension, irradiated with 2 W diode.
G IV	flooded with MTA suspension, irradiated with 4 W diode.
G V	flooded with MTA suspension, no laser irradiation.
G VI	flooded with distilled water, no laser irradiation.

### Laser irradiation

Canals of different groups were filled with one milliliter of either distilled water or MTA-suspension using size #25-gauge hypodermic needle (Monoject, Tyco Healthcare Kendall, USA). Canals were irradiated using Lasotronix laser diode device (Lasotronix 980 nm, Poland) in a continuous wave mode, via a 320 µm optic fiber. Power settings were adjusted to either 2-W (for groups; GI and G III) or 4-W (for groups; GII and G IV). The fiberoptic was introduced in the canal 1 mm shorter of the working length, activated and withdrawn coronally in a spiral manner within 5 s. The irradiation cycle was repeated 3 times with a total time of laser irradiation of 15 s for each canal with cooling interval of 5 s between each cycle. Excess solution left in canals was blotted out with a clean, dry paper point.

### Specimen preparation for microscopic examination

Root samples were split vertically using a double-sided diamond disc, then total separation was completed by mallet and chisel.

### Environmental scanning electron microscope (ESEM)

Samples were prepared for viewing under the environmental scanning electron microscope (ESEM) to evaluate root canal dentin topography and amount of surface particles at 1000X magnification (Prisma E, Thermofisher Co. Waltham, Massachusetts, USA).

### Qualitative and quantitative analysis

The mean value of open dentinal tubules (DT) was quantitatively evaluated per micrograph as a mean value of the gray scale, per a fixed and constant surface area 100 × 100 nm at the center of the micrograph, using an image analysis software (Image-J 1.54D, Wayne Rasband and Contributors, National Institutes of Health, USA). The selected area was copied, sharpened, and contrast-enhanced to optimize visibility. The threshold was then adjusted to ensure all dentinal tubule openings were incorporated. The image was subsequently converted into a binary high-contrast (black and white) format, where the mean pixel percent value was calculated. This value served as an indirect indicator of dentinal tubule opening, which is inversely proportional to the amount of dentine coverage. The assessment of the images was carried out by the blinded assessor A.A.S.

Moreover, five scale scoring system was employed to evaluate efficiency of covering root surface by a blinded investigator. The scoring system to evaluate the surface coverage followed the scoring system published in the literature as follows: [23]

1. All dentinal tubules were open,
2. A minor area was covered (more than 75% of the dentinal tubules remained open),
3. The dentine was partially covered (50–75% of the dentinal tubules remained open),
4. The dentine was largely covered (10–49% of the dentinal tubules remained open),
5. The dentine was fully covered (fewer than 10% of the dentinal tubules remained open)

### Statistical analysis

The Kolmogorov-Smirnov test has revealed the normal distribution of the data of image J analysis, therefore, one-way ANOVA test was used for statistical analysis at a significance level of 0.05, while Post Hoc test was used for comparison between subgroups. Regarding the data of the scoring, due to the non-parametric nature of the data, the Kruskal-Wallis test was used to determine the presence of statistically significant differences across groups. When significant differences were detected, pairwise comparisons were performed using the Mann-Whiteny to identify specific group differences. A significance level of *p* < 0.05 was considered for all analyses.

## Results

**Fig. 1:**
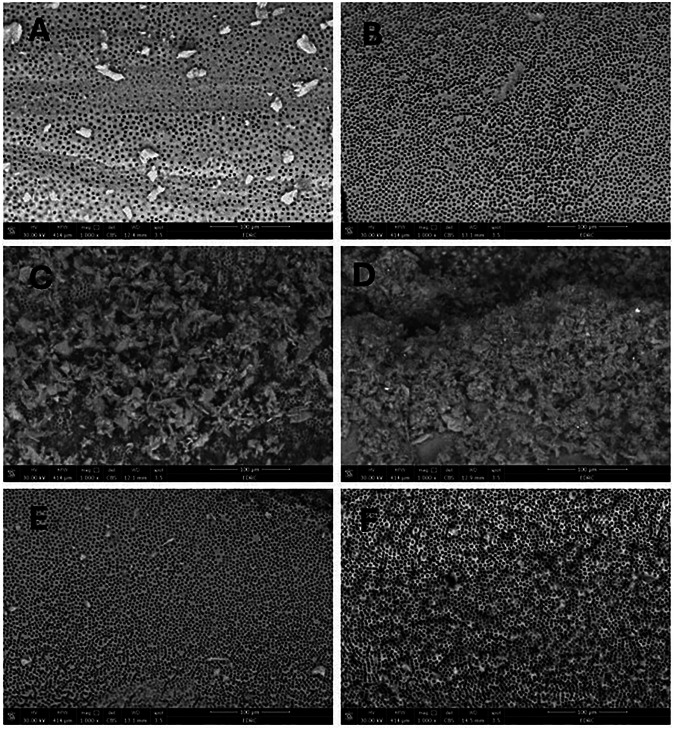
ESEM micrograph of the dentin surface at the root canal at magnification 1000X. Groups GI, II, III, IV, V, and VI are demonstrated from upper left till lower right respectively; the majority of the dentine surface is minimally covered **A**, the coalescence and diminish of dentinal tubules diameter **B**, majority of the dentine surface heavily covered **C, D**, sporadic precipitates on dentine surface **E**, and the normal dentine surface architecture in **F**.

The results revealed the potential of diode laser power to induce surface precipitation of MTA nanoparticles on root canal surface at laser power outputs of 2 W and 4 W as shown in Fig. 1C, D respectively. One-way ANOVA demonstrated significance among all the groups. The highest mean pixel percent values representing the open DT was displayed in the negative control group (G VI), where percent value reached 53.4, followed by GV, in which the mean percent value was 36. The least mean value was reported in G IV (3.4) followed by G III, with a statistically significant difference between both power outputs. The dentine surface was significantly covered with the solid phase of the suspension, where the precipitates were interconnected and interlaced in Fig. 1C, D. The irradiated distilled water samples in subgroups G I and G II demonstrated little precipitate as shown in Fig. 1A, B respectively. The effect of flooding the root surface with nano MTA suspension without subsequent laser irradiation in G V was presented in Fig. 1E. Normal dentine architecture with evident open dentinal tubules was presented in Fig. 1F. The results are shown in Table 2.

**Table 2:** Mean and standard deviation (SD) of pixel percent values of all groups.

Group	Mean ± SD	
G I	30.2 ± 5.1^a^	
G II	37.1 ± 8.1^a^	P < 0.001*
G III	16.7 ± 3^b^	
G IV	3.4 ± 3.1^c^	
G V	36 ± 4.6^a^	
G VI	53.4 ± 9.6^d^	

Different letters indicate statistically significant differences. Groups sharing the same letter are not significantly different from each other.

*Statistically Significant difference (*P* ≤ 0.05).

On the other hand, Regarding the scoring system, a statistically significant higher scores were revealed in the laser irradiated nanoMTA flooded canals (median value of 4) among all groups, however no statical significance was detected between the other 4 groups. Results are shown in Table 3.

**Table 3:** Median and range of scores of all groups.

Group	Median [Range]	
G I	2 [1-2]^a^	
G II	1[1-2]^a^	*P* < 0.001*
G III	4[3-5]^b^	
G IV	4[4-5]^b^	
G V	2[1-2]^a^	
G VI	1[1-2]^a^	

Different letters indicate statistically significant differences. Groups sharing the same letter are not significantly different from each other.

* Statistically Significant difference (*P* ≤ 0.05).

## Discussion

The present study explored the feasibility of using a 980-nm diode laser to induce surface precipitation of MTA nanoparticles in suspension on the dentin surface of root canals. This novel approach for coating dentin surfaces with nano-MTA shows promise for advancing endodontic treatments. Given MTA’s sealing properties and its ability to stimulate tissue regeneration [24, 25], it offers an innovative solution for addressing the challenges of dentin tubules’ sealing in root canal therapy. By inducing nano-level dentinal sealing, bacterial infiltration, and nano- leakage are expected to be reduced.

As the nanoscale of the particles significantly enhances their bioactivity [26], recent evidence advocated the nano-MTA based root canal sealers regarding sealing abilities, adaptability and antibacterial effects [27]. For these reasons, nano MTA was deemed the most appropriate choice. A 1:12 concentration of MTA nanoparticles in distilled water was selected based on pilot study, ensuring suspension stability for 30 s prior to sedimentation.

Dentinal wall coverage was assessed using environmental scanning electron microscopy (ESEM), chosen over scanning electron microscopes (SEM) to preserve sample integrity and provide accurate topographical analysis. While SEM provide high-quality, and detailed images of specimen topography, they may distort samples due to the ethanol dehydration and metal coating required [28]. ESEM, on the other hand, enables the analysis of specimens in a more natural state, often eliminating the need for conductive coating or dehydration, making it particularly suitable for topographical assessment in this study. Both qualitative scoring system and Image J quantitative analysis of micro images were carried out in alignment with the current literature [23, 28].

For this study, laser output power settings of 2 W and 4 W were selected. With appropriate calibration of laser equipment, thermal damage to surrounding periodontal tissues is minimal, as root canal temperature increases remain below 5 °C [29]. However, in this study, a 4-W power setting caused a noticeable rise in root surface temperature, enough to be felt at the fingertips.

The findings from the laser-irradiated experimental groups aligned with the well-documented effects of laser power on dentinal tubule occlusion [30], particularly evident with higher power settings. The results in the current study demonstrated that both diode laser power outputs tested successfully induced sintering of MTA-nanoparticles in suspension, resulting in a significant accumulation of surface-sintered particles. The photo-thermal energy delivered by the diode laser effectively facilitated the precipitation of the suspended hydrophilic calcium silicate gel-like particles, a phenomenon not observed in the irradiated distilled water groups which lacked suspended particles. Although Image J analysis showed a statistically significant difference favoring G IV over G III, this significance was not reflected in the scoring system. This finding supports the use of a lower power output for potential clinical benefits.

The substantially higher coverage scores of laser-irradiated nano-MTA suspension-treated root samples, compared to non-irradiated nano-MTA suspension flooded samples, demonstrated that thermal effects from the laser directly induced precipitation and root surface coverage, beyond what could be attributed to gravitational settling alone. This laser-induced precipitation aligns with findings from laser-induced precipitation observed in high-entropy alloys, though this study involved much higher power settings, up to 250 W, for metal alloy processing [31].

A likely explanation for the formation of a dispersible precipitate on the dentin surface after laser irradiation with distilled water at 2 and 4 W is the interaction between laser energy and trace minerals or dissolved gases in the distilled water. Additionally, laser irradiation may modify dentin’s mineral components and surface topography, resulting in a superficial precipitate layer from reprecipitated ablation debris [32].

Laser treatment induces chemical and compositional changes in dentin, including melting, recrystallization, and hydroxyapatite crystal growth, with surface alterations distinct from those produced by high-temperature oven sintering [33]. Such topographical and compositional changes could partially explain such phenomena.

The chemical and topographical heterogeneity of the substrate surface leads to contact angle hysteresis. On such heterogeneous surfaces, like dentin, a range of contact angles is observed rather than a single value as predicted by Young’s equation for ideal surfaces [34]. This variability likely accounts for the non-uniform overlapping nature of the MTA-nanoparticle precipitation observed in ESEM micrographs, as uniform precipitation would only be expected if the substrate had a homogenous surface.

This pioneering research demonstrates the potential of diode laser power for dentin surface coverage through laser sintering of MTA nanoparticles in suspension, offering a new direction for studies in this area. However, some limitations are noted. the lack of real-time temperature monitoring during irradiation, restricting insights into thermal effects on surrounding tissues. Additionally, the study’s focus on a specific nano-MTA suspension in distilled water restricts the generalizability of its findings to other solvents or concentrations. Future research should thus investigate diverse solvents and concentrations. Further studies should also examine the ability of sintered MTA particles, especially in nano-MTA suspensions, to penetrate dentinal tubules and evaluate the mineral composition of the resulting surface layer over different application durations.

## Conclusion

The 980-nm diode laser effectively promotes MTA nanoparticle precipitation on root canal surfaces, enhancing dentinal tubule occlusion and sealing capacity. This approach shows promise for improving root canal therapy outcomes. Further research is needed to optimize laser parameters, refine MTA suspension ratios, and evaluate clinical efficacy.

## Data Availability

The datasets utilized and analyzed in this study can be obtained from the corresponding author upon reasonable request.
